# Differentially Expressed Genes and Signature Pathways of Human Prostate Cancer

**DOI:** 10.1371/journal.pone.0145322

**Published:** 2015-12-18

**Authors:** Jennifer S. Myers, Ariana K. von Lersner, Charles J. Robbins, Qing-Xiang Amy Sang

**Affiliations:** 1 Department of Chemistry and Biochemistry, Florida State University, Tallahassee, Florida, United States of America; 2 Institute of Molecular Biophysics, Florida State University, Tallahassee, Florida, United States of America; Stony Brook University, UNITED STATES

## Abstract

Genomic technologies including microarrays and next-generation sequencing have enabled the generation of molecular signatures of prostate cancer. Lists of differentially expressed genes between malignant and non-malignant states are thought to be fertile sources of putative prostate cancer biomarkers. However such lists of differentially expressed genes can be highly variable for multiple reasons. As such, looking at differential expression in the context of gene sets and pathways has been more robust. Using next-generation genome sequencing data from The Cancer Genome Atlas, differential gene expression between age- and stage- matched human prostate tumors and non-malignant samples was assessed and used to craft a pathway signature of prostate cancer. Up- and down-regulated genes were assigned to pathways composed of curated groups of related genes from multiple databases. The significance of these pathways was then evaluated according to the number of differentially expressed genes found in the pathway and their position within the pathway using Gene Set Enrichment Analysis and Signaling Pathway Impact Analysis. The “transforming growth factor-beta signaling” and “Ran regulation of mitotic spindle formation” pathways were strongly associated with prostate cancer. Several other significant pathways confirm reported findings from microarray data that suggest actin cytoskeleton regulation, cell cycle, mitogen-activated protein kinase signaling, and calcium signaling are also altered in prostate cancer. Thus we have demonstrated feasibility of pathway analysis and identified an underexplored area (Ran) for investigation in prostate cancer pathogenesis.

## Introduction

Prostate cancer is the second most diagnosed cancer among American men, with over 220,000 new cases predicted in 2015 [1]. Prostate-specific antigen (PSA) has been the cornerstone of prostate cancer screening for decades. However PSA is not an ideal biomarker and widespread use of PSA-screening is falling out of favor [2–4]. Reliance on PSA screening is problematic because false positives result from benign prostatic hyperplasia or prostatitis and because PSA fails to discriminate indolent disease, leading to overdiagnosis. The expansion of genomic and proteomic technology and methodology has improved the characterization of tumor biology, driving the search for more accurate cancer biomarkers. Gene and protein expression differences between normal and malignant prostate tissues have been well documented and serve as a pool for putative diagnostic, prognostic, and risk stratification biomarkers [5–24]. Gene mutations, epigenetic changes, and microRNA expression changes that occur in cancer initiation and progression have also been studied with the goal of biomarker discovery [25–29]. Yet there remain several substantial obstacles in biomarker implementation. Low reproducibility across laboratories, differences in experimental platforms and techniques, the inherent heterogeneity of prostate cancer, and insignificant clinical utility or small gains in sensitivity and specificity beyond PSA hampers the identification, validation, and implementation of biomarkers [30–35].

Previous work has focused on the selection and validation of individual genes as biomarkers. Yet the heterogeneity of prostate cancer makes it extremely unlikely to find a single gene that is a representative marker [36]. Screening panels formed by the combination of multiple genes have been used to increase predictive power for cancer detection, recurrence, relapse, and survival beyond the use of PSA or Gleason score alone [37–40]. The success of the biomarker panel approach is evidenced by the commercial launch of several screening tests which have found clinical usefulness: ProMark [41], Oncotype DX [42], Prolaris [43], and Decipher [44]. These panels may be pulled from molecular classifications studies that use differential expression to craft a signature for cancer.

However molecular classifications and gene signatures are not always stable in the sense that multiple signatures can be found for cancers. Large discrepancies between lists of differentially expressed genes (DEGs) from microarray data have been highlighted [45]. In some cases the overlap between microarray datasets was as low as 5% [46]. So for each set of DEGs, a different signature could be found. Thus biomarkers selected from these lists would perform with varying degrees of success. Taking the list of DEGs and correlating them to a prognostic marker may generate a more useful putative biomarker pool because then only genes correlated with prognosis would comprise the molecular signature. However, Ein-Dor *et al*. showed that in breast cancer, there was no single, unique set of genes that predicted survival because altering the patient population could produce multiple sets of genes of equal prognostic ability in predicting survival [33]. Furthermore, correlation with survival was not required for prognostic ability [33]. So it is likely that many panels exclude a number of other genes that could be potential biomarkers because the panel was derived from one body of samples (although it may be large) and considered only strongest correlations.

An alternative approach is pathway-based analysis. In pathway analysis, a collection of related genes from the same pathway or network of interaction is assessed instead of examining a group of potentially unrelated genes that optimize sensitivity and selectivity of diagnosis or prognosis. There is increased overlap between data at the pathway level compared to overlap between lists of DEGs [46, 47]. Pathway analysis does not neglect the cooperative nature of genes and considers that oftentimes genes involved in the same process are often deregulated together. By looking at the pathway, minor variations in instrumentation or method are less likely to impact results, leading to more consistent results across different sets of data [48]. Thus the pathway approach yields more robust results, improves disease classification, and may reveal novel insights about a disease [49–51]. One type of pathway analysis starts with a differentially expressed gene and correlates the expression of genes involved in the same pathway or similar process with a particular diagnostic or prognostic outcome [52–54]. A similar iteration starts with a pathway of known importance in cancer initiation or progression and evaluates the prognostic power of its individual components. This has been done for the mitogen-activated protein kinase (MAPK) pathway [55], Akt [56], mTOR pathway [57, 58], Toll-like receptor signaling pathway [59], and other oncogene signatures [60].

In this paper, comprehensive gene expression in human prostate cancer was characterized using an unbiased pathway approach. Next generation sequencing was used to obtain a profile of the differences in RNA expression between human tumors and non-malignant tissue from patients. Pathway analysis included Gene Set Enrichment Analysis and Signaling Pathway Impact Analysis. Two pathways were significantly associated with human prostate tumors—“Ran regulation of mitotic spindle formation” pathway and “transforming growth factor-beta (TGF-β) signaling” pathway.

## Materials and Methods

### RNA sequencing data

Level 3 de-identified data for prostate cancer samples and all available non-malignant samples from these prostate cancer patients was downloaded from The Cancer Genome Atlas (TCGA) data portal (https://tcga-data.nci.nih.gov). Level 3 describes data that has been processed and aggregated to give gene expression signals for a sample. For each sample, the data contains expression counts for up to 20,531 coding and non-coding RNA transcripts plus clinical information such as age, stage, Gleason score, PSA level, and race/ethnicity. Before analysis, tumor and non-malignant samples were randomly pulled to achieve an age- and stage-matched pool of 225 samples (S1 Table). A total of 173 prostate cancer samples and 52 non-malignant samples from 204 unique patients were analyzed. The patient clinical information is presented in Table 1.

**Table 1:** Prostate cancer patient clinical information from TCGA.

Characteristics	Samples (n = 225)	Tumor (n = 173)	Non-Malignant (n = 52)	Fisher’s Exact Test P-value
**Age**				
< 65	155	121	34	0.609
≥ 65	70	52	18	
**Pathological T stage**				
T1	0	0	0	0.649
T2	113	84	29	
T3	103	82	21	
T4	8	6	2	
Unspecified	1	1	0	
**Race**				
White	92	50	42	0.701
Black	7	3	4	
Unspecified	126	120	6	
**Ethnicity**				
Not Hispanic	96	51	45	
Unspecified	129	122	7	
**Gleason Score**				
≤ 6	24	19	5	0.00168
7	129	89	40	
8–10	72	65	7	

### Differential Gene Expression

The R programming environment (version 3.1.2) [61] was used to process raw data, perform statistical calculations, and perform differential expression analysis. After age- and stage-matching, 393 transcripts were removed because they lacked expression in the 225 samples comprising the dataset. The RNA counts for the remaining 20,138 transcripts were rounded to the nearest whole number and compiled into a matrix to build the dataset. The magnitude of expression changes relative to non-malignant samples was also calculated by taking the base 2 logarithm of the tumor/non-malignant mean expression ratio. For genes with no expression in either the tumor or non-malignant samples, the log_2_ fold changes were adjusted by adding one to each mean and then calculating the ratio. All log_2_ values quoted are values after any such adjustments. Negative fold changes indicated down-regulation in tumor samples whereas positive values indicated up-regulation. The R package DESeq2 (version 1.6.3) [62] was used to identify DEGs in the TCGA patient RNA data. The computing was done on the Florida State University High Performance Computing Cluster. DESeq2 returned a P-value determined by Wald statistics and an adjusted P-value (Q-value) to correct for multiple comparisons testing using the Benjamini-Hochberg method to determine the false discovery rate (FDR). DEGs were defined as genes different with a FDR less than 1% (Q < 0.01).

To evaluate the significance of the identified DEGs, analyses were conducted to search for overrepresented pathways, gene set enrichment, and signaling pathway impact. First, overrepresented elements were identified among the DEGs. The Protein ANalysis THrough Evolutionary Relationships (PANTHER) Classification System and analysis tools were used to categorize DEGs by PANTHER protein class, Gene Ontology (GO) Molecular Function, and GO Biological Process to then determine if any of these classes or GO terms were overrepresented [63]. The PANTHER Overrepresentation Test (release 20150430) was used to search the data against the PANTHER database (PANTHER version 10.0 Released 2015-05-15) and the GO database (Released 2015-05-09) to identify either protein classes or GO annotations overrepresented in our data when compared to a reference human genome. P-values were adjusted using a Bonferroni correction.

### Pathway Analysis

Gene Set Enrichment Analysis (GSEA) [64] was used to identify groups of genes enriched in either the tumor or non-malignant condition. The GSEA analysis tool (version 2.2.0) was downloaded from the Broad Institute website (http://www.broadinstitute.org/gsea/index.jsp). Curated gene sets of BioCarta and Reactome pathways were downloaded from the Broad Institute’s Molecular Signatures Database. An additional gene set was constructed from Kyoto Encyclopedia of Genes and Genomes (KEGG) pathways [65]. Pathways with the least relevance to prostate cancer were excluded. The KEGG pathways included in the analysis are listed in the Supporting Information (S2 Table). The entire RNA expression count matrix was loaded into the GSEA application without limiting the input to only DEGs. Both small (< 5 genes) and large (> 500 genes) gene sets were excluded from the analysis.

Signaling Pathway Impact Analysis (SPIA) was used to assess the importance of enriched pathways in terms of their impact and ability to activate or inhibit a pathway [66]. SPIA analysis was accomplished using the R package “SPIA” (version 2.18.0) [67]. Entrez IDs, log_2_ fold changes, and Q-values for all genes were compiled. The differential expression cut-off used in the SPIA algorithm was based on the FDR-adjusted Q-value. The analysis was run using the same tailored list of pathways as used in GSEA (S2 Table) and updated versions of these pathways were download prior to running the analysis (accessed 7/29/2015).

## Results

**Fig 1:**
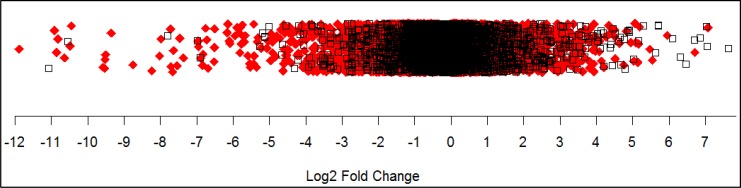
Magnitude of gene expression differences between tumor and non-malignant human prostate cancer samples. In this one-dimensional scatter plot the magnitude of gene expression changes represented by log_2_ fold ratios are shown. Each point represents a gene or transcript. Significantly differentially expressed genes and transcripts are shown as solid red diamonds.

Using a 1% FDR (Q <0.01), DESeq2 analysis marked 11,115 genes and transcripts as statistically different between tumor samples and non-malignant samples in our TCGA dataset (S3 and S4 Tables). This covers 55% of the genes and transcripts sequenced. The number of down-regulated genes and transcripts totaled 5,379 and the number of up-regulated genes and transcripts totaled 5,736. Overall the largest changes observed were in the down-regulation of genes and transcripts (Fig 1). The magnitude of the up-regulation of genes and transcripts was smaller than the magnitude of down-regulated genes and the range of expression was also smaller. The twenty most down-regulated and the twenty most up-regulated genes are presented in Table 2 and Table 3.

**Table 2:** Twenty largest decreases in RNA expression between prostate tumor and non-malignant TCGA samples.

Gene Symbol	Name	Log_2_ Fold Change	P-value	Q-value
*WFDC9*	Protein WFDC9	-11.89	1.98E-04	4.30E-04
*DEFB125*	Beta-defensin 125	-10.91	4.30E-04	8.89E-04
*EDDM3B*	Epididymal secretory protein E3-beta	-10.85	4.64E-09	1.96E-08
*PAEP*	Glycodelin	-10.82	3.47E-16	3.78E-15
*SEMG2*	Semenogelin-2	-10.64	1.98E-63	2.36E-60
*PATE4*	Prostate and testis expressed protein 4	-10.48	2.26E-55	1.54E-52
*EDDM3A*	Epididymal secretory protein E3-alpha	-10.45	5.77E-13	4.05E-12
*CRISP1*	Cysteine-rich secretory protein 1	-9.58	1.17E-25	5.02E-24
*PATE1*	Prostate and testis expressed protein 1	-9.53	3.27E-27	1.76E-25
*DEFB127*	Beta-defensin 127	-9.52	1.06E-04	2.41E-04
*AQP2*	Aquaporin-2	-9.50	1.94E-57	1.69E-54
*TMEM114*	Transmembrane protein 114	-9.35	1.19E-15	1.21E-14
*GRXCR1*	Glutaredoxin domain-containing cysteine-rich protein 1	-8.75	5.95E-19	9.64E-18
*SPINT3*	Kunitz-type protease inhibitor 3	-8.23	2.11E-24	7.48E-23
*CLDN2*	Claudin-2	-8.02	2.11E-75	6.72E-72
*SULT2A1*	Bile salt sulfotransferase	-7.98	9.41E-20	1.70E-18
*SPINK2*	Serine protease inhibitor Kazal-type 2	-7.71	5.75E-71	8.46E-68
*POU3F3*	POU domain, class 3, transcription factor 3	-7.70	4.68E-17	5.77E-16
*LCN1*	Lipocalin-1	-7.66	4.18E-08	1.56E-07
*PATE3*	Prostate and testis expressed protein 3	-7.63	3.33E-25	1.32E-23

Log_2_ fold change describes malignant expression relative to non-malignant expression. P-value is determined by DESeq2 using Wald Statistics and Q-value is the false discovery rate-adjusted P-value.

**Table 3:** Twenty largest increases in RNA expression between prostate tumor and non-malignant TCGA samples.

Gene Symbol	Name	Log_2_ Fold Change	P-value	Q-value
*ANKRD30A*	Ankyrin repeat domain-containing protein 30A	7.08	5.95E-10	2.82E-09
*FEZF2*	Fez family zinc finger protein 2	6.71	1.89E-06	5.59E-06
*C6orf10*	Uncharacterized protein C6orf10	5.96	2.59E-06	7.52E-06
*FOXG1*	Forkhead box protein G1	5.54	2.53E-04	5.41E-04
*GC*	Vitamin D-binding protein	5.47	4.70E-04	9.67E-04
*VAX1*	Ventral anterior homeobox 1	5.19	3.83E-12	2.41E-11
*SSX2*	Protein SSX2	5.16	4.52E-03	7.92E-03
*FGB*	Fibrinogen beta chain	5.14	1.52E-03	2.88E-03
*SLC45A2*	Membrane-associated transporter protein	5.09	1.10E-51	5.99E-49
*SPINK1*	Pancreatic secretory trypsin inhibitor	5.07	3.29E-12	2.08E-11
*HOXC12*	Homeobox protein Hox-C12	5.03	1.44E-07	4.96E-07
*SCN1A*	Sodium channel protein type 1 subunit alpha	4.96	5.38E-03	9.31E-03
*LOC284661*	Uncharacterized non-coding RNA	4.84	4.89E-06	1.36E-05
*TFDP3*	Transcription factor Dp family member 3	4.76	2.00E-03	3.72E-03
*B3GNT6*	UDP-GlcNAc:betaGal beta-1,3-N-acetylglucosaminyltransferase 6	4.64	5.49E-22	1.40E-20
*FOXB2*	Forkhead box protein B2	4.52	2.14E-18	3.19E-17
*NR2E1*	Nuclear receptor subfamily 2 group E member 1	4.51	1.21E-15	1.23E-14
*XAGE1E*	X antigen family, member 1E	4.51	4.00E-03	7.07E-03
*TBX10*	T-box transcription factor TBX10	4.43	6.47E-17	7.81E-16

Log_2_ fold change describes malignant expression relative to non-malignant expression. P-value is determined by DESeq2 using Wald Statistics and Q-value is the false discovery rate-adjusted P-value.

### Classification and Overrepresentation Analysis

**Fig 2:**
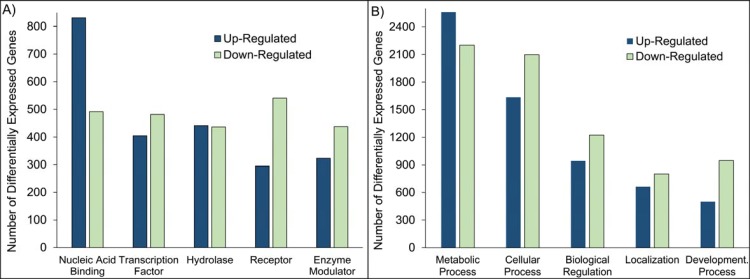
Functional Classification of Differentially Expressed Genes in Human Prostate Cancer According to PANTHER Protein Class (A) and Biological Process Gene Ontology Terms (B). (A) “Nucleic Acid Binding” includes RNA and DNA binding, nucleases, and helicases. “Transcription Factor” includes zinc finger, helix-turn-helix, high mobility group box, basic helix-loop-helix, and basic leucine zipper transcription factors; cofactors; and nuclear hormone receptors. “Hydrolase” refers to proteases, phosphatases, esterases, lipases, deaminases, phosphodiesterases, glycosidases, deacetylases, pyrophosphatases, glucosidases, galactosidases, and amylases. “Receptor” includes protein kinase receptors, nuclear hormone receptors, cytokine receptors, ligand-gated ion channels, and G-protein coupled receptors. “Enzyme Modulator” includes G protein, kinase, phosphatase, and protease modulators. (B) “Metabolic Process” features carbohydrate, cellular amino acid, lipid, protein, and nucleobase-containing compound metabolism; and the tricarboxylic acid cycle. “Cellular Process” categories are cell-cell signaling, cell cycle, growth and proliferation, cell component movement, and cytokinesis. “Biological Regulation” includes the regulation of apoptosis, metabolism, cell cycle, translation, catalytic activity, and homeostasis. “Developmental Process” categories are system, ectoderm, mesoderm, and endoderm development; cell differentiation; death; anatomical structure morphogenesis; embryo development; sex determination; and pattern specification processes. “Localization” includes transport proteins, protein and RNA localization processes.

The 11,115 DEGs were grouped according to PANTHER protein class, GO Molecular Function and GO Biological Process annotations. A total of 6,254 DEGs had either PANTHER protein class, GO Biological Process, or GO Molecular Function annotations and were further classified. Grouping by protein class and GO Biological Process categories proved to be the most informative (Fig 2). The complete classifications can be found in the Supporting Information (S5 Table). The DEGs represent a wide spectrum of protein classes involved in a broad array of processes. The “Nucleic Acid Binding” PANTHER protein class includes both RNA and DNA binding proteins, nucleases, and helicases. The “Transcription Factor” protein class is sub-categorized by structural motif and also contains cofactors and nuclear hormone receptors. Proteases and phosphatases are found within the “Hydrolase” protein class. The types of “Receptor” included are protein kinase receptors, nuclear hormone receptors, cytokine receptors, ligand-gated ion channels, and G-protein coupled receptors. The “Enzyme Modulator” category features G protein, kinase, phosphatase, and protease modulators. Interestingly, the categories were generally not predominantly populated by down-regulated or up-regulated genes or transcripts. For all protein classes except the “Nucleic Acid Binding” class, DEGs were evenly distributed across tumor and non-malignant samples. In the “Nucleic Acid Binding” protein class, there were nearly one and half times as many up-regulated genes as down-regulated. The abundance of nucleic acid binding genes suggests altered transcriptional activity in tumor samples.

The two most abundant GO Biological Process groups—“Metabolic Process” and “Cellular Process”—are not surprising because these contains genes are involved in the most basic of life processes. In fact, metabolic changes have been widely documented in tumors [68–70]. The increased energetic and biosynthetic needs of proliferating cancer cells are often met through metabolic dysregulation [71–73]. The heading “Metabolic Process” includes carbohydrate metabolism, cellular amino acid metabolism, lipid metabolism, nucleobase-containing compound metabolism, protein metabolism, and the tricarboxylic acid cycle. “Cellular Process” includes cell-cell signaling, cell cycle, growth and proliferation, cell component movement, and cytokinesis. “Biological Regulation” includes the regulation of apoptosis, metabolism, cell cycle, translation, catalytic activity, and homeostasis. The category “Developmental Process” incorporates system, ectoderm, mesoderm, and endoderm development, as well as cell differentiation, death, anatomical structure morphogenesis, embryo development, sex determination, and pattern specification processes. “Localization” refers to general transport proteins and specific protein and RNA localization processes.

PANTHER’s overrepresentation statistic was used to calculate the probability that the highly populated protein classes and GO groupings among the DEGs would occur by random chance. Indeed, many of the most abundant categories are overrepresented in the data when compared to a reference genome (Table 4). The three most abundant protein classes—“Nucleic Acid Binding”, “Transcription Factor”, and “Hydrolase”—were enriched along with the classes “Transferase” and “Transporter”. The five most populated GO Biological Processes were also enriched: “Metabolic Process”, “Cellular Process”, “Biological Regulation”, “Localization”, and “Developmental Process”. The “Multicellular Organism Process”, “Biological Adhesion”, “Cellular Component Organization or Biogenesis”, and “Immune System Process” GO Biological Processes were also enriched. Finally, five of the top six GO Molecular Functions were enriched: “Binding”, “Catalytic Activity”, “Nucleic Acid Binding Transcription Factor Activity”, “Transporter Activity”, and “Structural Molecule Activity”.

**Table 4:** Overrepresented PANTHER protein class and GO ontology categories in TCGA data from malignant and non-malignant prostate.

	P-value
**PANTHER Protein Class**	
RNA binding protein (Nucleic Acid Binding)	9.08E-05
Ribosomal protein (Nucleic Acid Binding)	4.67E-04
Transcription factor	2.04E-02
Transferase	3.41E-04
Hydrolase	5.75E-04
Transporter	3.65E-03
**GO-Biological Process**	
Sensory perception of chemical stimulus (Multicellular Organism Process)	2.96E-10
Protein metabolic process (Metabolic Process)	6.82E-09
Nucleobase-containing compound metabolic process (Metabolic Process)	1.64E-08
RNA metabolic process (Metabolic Process)	6.87E-06
Nervous system development (Developmental Process)	1.48E-03
Cellular protein modification process (Metabolic Process)	1.90E-03
Translation (Metabolic Process)	2.81E-03
Natural killer cell activation (Immune System Process)	4.06E-03
DNA-dependent transcription (Metabolic Process)	7.61E-03
Ion transport (Localization)	1.81E-02
Protein phosphorylation (Metabolic Process)	3.31E-02
Cellular component morphogenesis (Cellular Component Organization or Biogenesis)	3.85E-02
Cellular component organization (Cellular Component Organization or Biogenesis)	7.58E-04
Cell communication (Cellular Process)	2.57E-02
Biological regulation	3.03E-05
Biological adhesion	4.67E-02
**GO-Molecular Function**	
Transferase activity (Catalytic Activity)	1.67E-06
Hydrolase activity (Catalytic Activity)	2.87E-04
Kinase activity (Catalytic Activity)	7.73E-03
Protein binding (Binding)	2.99E-03
DNA binding (Binding)	4.19E-03
Transmembrane transporter activity (Transporter Activity)	3.37E-03
Sequence-specific DNA binding transcription factor activity (Nucleic Acid Binding Transcription Factor Activity)	1.52E-02
Structural constituent of ribosome (Structural Molecule Activity)	3.59E-02

Overrepresentation was determined by calculating the probability that the number of differentially expressed genes belonging to a particular category is larger or smaller than what would be expected based on a reference human genome. P-values are adjusted using a Bonferroni correction.

### Gene Set Enrichment Analysis

One limitation of a class or pathway overrepresentation analysis is that it does not indicate which condition is associated with the overrepresentation; GSEA does. Expressed genes were ranked by their correlation with the malignant phenotype and then this list was compared to sets of genes in a pathway, linking pathway enrichment to a phenotype. The more highly-correlated genes in a gene set, the higher the significance of that gene set. The gene sets with the highest normalized enrichment scores are presented in Table 5 and other results are listed in the Supporting Information (S7 Table). The FDR cutoff was set at 25% to maximize hypothesis generation. Only one pathway was enriched in the tumor samples, the “RanMS pathway” which includes the genes that regulate the formation of the mitotic spindle during cell division. Ten genes in our list of DEGs belonged to this pathway, each contributing to its enrichment in the malignant phenotype (Table 6). All ten were differentially expressed and up-regulated in the malignant samples. The remaining pathways were enriched in the non-malignant phenotype. The most significant pathway enriched in the non-malignant phenotype was the “calcium signaling” pathway. Enrichment of the calcium signaling pathway was due to 81 DEGs and 19 other genes or transcripts (S8 Table). Also enriched in the non-malignant phenotype were several other signaling pathways (oxytocin, prolactin, cAMP, MAPK, cGMP-PKG, TGF-β, Hippo, and Ras) and pathways related to cell-cell and cell-matrix adhesion (extracellular matrix-receptor interaction, actin cytoskeleton regulation, proteoglycans, and focal adhesion).

**Table 5:** Significant gene sets enriched in malignant and non-malignant prostate with the largest normalized enrichment scores.

Gene Set	ES	NES	P-value	Q-value
BioCarta: RanMS pathway	0.827	1.652	4.02E-03	2.05E-01
KEGG: Calcium signaling pathway	-0.456	-1.714	4.89E-03	5.75E-02
KEGG: Basal cell carcinoma	-0.482	-1.647	6.59E-03	6.10E-02
KEGG: Oxytocin signaling pathway	-0.443	-1.603	1.66E-02	6.26E-02
KEGG: Thyroid hormone synthesis	-0.466	-1.652	1.19E-02	6.34E-02
KEGG: Signaling pathways regulating pluripotency of stem cells	-0.432	-1.596	4.56E-03	6.35E-02
KEGG: Prolactin signaling pathway	-0.461	-1.605	4.52E-03	6.44E-02
KEGG: Pathways in cancer	-0.433	-1.624	8.77E-03	6.49E-02
KEGG: ECM-receptor interaction	-0.517	-1.633	3.76E-02	6.52E-02
KEGG: cAMP signaling pathway	-0.434	-1.719	0.00E+00	6.63E-02
KEGG: MAPK signaling pathway	-0.417	-1.614	2.28E-03	6.78E-02
KEGG: Regulation of actin cytoskeleton	-0.449	-1.606	8.99E-03	6.84E-02
KEGG: Phosphatidylinositol signaling pathway	-0.517	-1.653	4.38E-03	6.93E-02
KEGG: cGMP-PKG signaling pathway	-0.469	-1.677	1.43E-02	7.12E-02
KEGG: TGF-beta signaling pathway	-0.513	-1.654	1.11E-02	7.73E-02
KEGG: Focal adhesion	-0.517	-1.725	1.61E-02	7.87E-02
KEGG: Hippo signaling pathway	-0.482	-1.733	0.00E+00	9.65E-02
KEGG: Proteoglycans in cancer	-0.492	-1.740	0.00E+00	1.38E-01
KEGG: Ras signaling pathway	-0.460	-1.760	2.23E-03	2.13E-01
BioCarta: p38 MAPK pathway	-0.527	-1.584	8.15E-03	2.48E-01

ES = enrichment score, NES = normalized enrichment score, Q-value = false discovery rate-adjusted P-value. Positive enrichment scores correspond to enrichment in the malignant samples. Negative enrichment scores correspond to enrichment in the non-malignant samples.

**Table 6:** Differentially Expressed Ran-Mitotic Spindle Pathway Components in Human Prostate Cancer.

Ran regulation of mitotic spindle formation pathway			
Gene Name	Symbol	Expression	Role
GTP-binding nuclear protein Ran	*RAN*	↑	GTPase; nuclear transport; formation of mitotic spindle [74]
Regulator of chromosome condensation	*RCC1*	↑	Guanine nucleotide exchange factor of Ran, produces a RanGTP gradient around chromosomes. [75]
Ran GTPase-activating protein 1	*RANGAP1*	↑	Accelerates RanGTP hydrolysis, helps maintain RanGTP gradient around chromosomes. [75]
Ran binding protein 1	*RANBP1*	↑	Regulates activity of RCC1 and RANGAP [76, 77]
Importin subunit alpha-1	*KPNA2*	↑	Nuclear import; KPNB1 adapter protein [78]
Importin subunit beta-1	*KPNB1*	↑	Nuclear import; docking platform [79, 80]
Targeting protein for Xklp2	*TPX2*	↑	Spindle assembly factor; microtubule nucleation, separation of bipolar mitotic spindle [81, 82]
Nuclear mitotic apparatus protein 1	*NUMA1*	↑	Spindle assembly factor; Establishes, maintains mitotic spindle poles. [83]
Kinesin-like protein KIF15	*KIF15*	↑	Spindle assembly factor; Bipolar spindle maintenance, elongation [82]
Aurora kinase A	*AURKA*	↑	Centrosome maturation, separation, and centrosomal microtubule stabilization and nucleation. [84]

**↑** = up-regulated expression, **↓** = down-regulated expression

### Signaling Pathway Impact Analysis

SPIA considers whether or not the DEGs found in a pathway have a meaningful impact within that pathway and thus addresses the topology of DEGs in pathways [66]. In other words, pathway significance is partly dependent on if the number of DEGs observed in a pathway is larger than that observed by random chance. This is captured in the probability of overrepresentation. Pathway significance is also partly based on whether DEGs in a particular pathway are at crucial junctions and can thus perturb the pathway. This is the probability of perturbation. These two probabilities are combined into a global probability which is adjusted by the false discovery rate. This adjusted metric was used to rank the impact of the pathways. Many of the same pathways were identified as significant in both GSEA and SPIA analysis (Table 7). In fact, the 8 most significant pathway results from SPIA were all significantly enriched in GSEA. However, only the “calcium signaling” pathway was highly ranked in both analyses. The only pathway activated in the malignant condition was the “TGF-β signaling” pathway (Table 8). The other pathways were all inhibited in the malignant condition. Similar to GSEA results, several signaling pathways (oxytocin, cAMP, MAPK, cGMP-PKG, TGF-β, Hippo, Rap1, ErbB, and Ras) and pathways related to cell-cell and cell-matrix adhesion (proteoglycans, focal adhesion, and actin cytoskeleton regulation) were impacted. Images of the pathways with DEGs highlighted can be accessed in the Supporting Information (S9 Table).

**Table 7:** Significantly impacted pathways in human prostate cancer as determined by SPIA.

Name	NDE/pSize	pNDE	pPERT	pG	pGFdr
**Proteoglycans in cancer**	140/201	1.75E-05	5.00E-06	2.11E-09	1.86E-07
**Hippo signaling pathway**	112/153	3.12E-06	1.60E-02	8.90E-07	3.91E-05
**Pathways in cancer**	257/398	7.77E-05	2.00E-03	2.59E-06	7.60E-05
**Focal adhesion**	144/207	1.48E-05	2.10E-02	4.97E-06	1.09E-04
**cGMP-PKG signaling pathway**	118/167	2.80E-05	3.07E-01	1.09E-04	1.92E-03
**Calcium signaling pathway**	115/180	1.08E-02	4.00E-03	4.78E-04	7.01E-03
**Ras signaling pathway**	144/225	4.36E-03	2.90E-02	1.26E-03	1.59E-02
**TGF-β signaling pathway**	60/80	1.95E-04	8.54E-01	1.62E-03	1.78E-02
Chronic myeloid leukemia	55/73	2.93E-04	8.74E-01	2.37E-03	2.32E-02
**Basal cell carcinoma**	36/55	8.03E-02	6.00E-03	4.16E-03	3.59E-02
**Regulation of actin cytoskeleton**	135/213	9.11E-03	6.80E-02	5.20E-03	3.59E-02
ErbB signaling pathway	60/87	5.98E-03	1.07E-01	5.34E-03	3.59E-02
Glioma	48/65	1.50E-03	4.51E-01	5.60E-03	3.59E-02
Small cell lung cancer	58/86	1.38E-02	5.00E-02	5.71E-03	3.59E-02
**Oxytocin signaling pathway**	105/157	1.82E-03	4.53E-01	6.69E-03	3.87E-02
**MAPK signaling pathway**	152/254	7.51E-02	1.30E-02	7.75E-03	3.87E-02
Cell cycle	85/124	1.60E-03	6.41E-01	8.07E-03	3.87E-02
MicroRNAs in cancer	97/149	8.72E-03	NA	8.72E-03	3.87E-02
**cAMP signaling pathway**	132/200	1.15E-03	9.75E-01	8.76E-03	3.87E-02
Rap1 signaling pathway	130/211	3.42E-02	3.30E-02	8.80E-03	3.87E-02

NDE = number of differentially expressed elements, pSize = pathway size, pNDE = overrepresentation probability, pPERT = perturbation probability, pG = global probability, pGFdr = false discovery rate-adjusted global probability. Bold pathways were also significant by Gene Set Enrichment Analysis.

**Table 8:** Components of the TGF-β Signaling Pathway Differentially Expressed in Human Prostate Cancer.

TGF-β Signaling Pathway			
Gene Name	Symbol	Expression	Role
Transforming growth factor β-2	*TGF-β2*	↓	Cytokine growth factor [85]
Transforming growth factor β-3	*TGF-β3*	↓	Cytokine growth factor [85]
TGF-β receptor type I	*TGFBR1*	↓	transmembrane serine/threonine kinase [86]
TGF-β receptor type II	*TGFBR2*	↓	transmembrane serine/threonine kinase [86]
TGF-β receptor type III	*TGFBR3*	↓	non-signaling receptor, presents TGF-β ligands to TGFBR2 [86]
Latent-transforming growth factor β-binding protein 1	*LTBP1*	↓	maintains latency of TGF-β [87]
Mothers against decapentaplegic homolog 2	*SMAD2*	↓	receptor SMAD for TGFBR1 [88]
Mothers against decapentaplegic homolog 3	*SMAD3*	↓	receptor SMAD for TGFBR1 [88]
Mothers against decapentaplegic homolog 4	*SMAD4*	↓	complexes with receptor SMADs before nuclear translocation [88]
Mothers against decapentaplegic homolog 7	*SMAD7*	↓	blocks phosphorylation of SMAD 2/3 [89]
E3 ubiquitin-protein ligase RBX1	*RBX1*	↑	In complex with CUL1 degrades SMAD2/3 [90]
Cullin-1	*CUL1*	↓	In complex with RBX1 degrades SMAD2/3 [90]
Retinoblastoma-like protein 1	*RBL1*	↓	E2F4/5 corepressor of *myc* [91]
Transcription factor E2F4	*E2F4*	↓	*myc* transcription factor [91]
Transcription factor E2F5	*E2F5*	↑	*myc* transcription factor [91]
Myc proto-oncogene protein	*MYC*	↑	Influences cell growth, cell cycle, apoptosis, metabolism, energy production, DNA replication and RNA stability and splicing [92]
Sp1 Transcription factor	*SP1*	↓	Transcription factor regulating growth factors, DNA synthesis regulators, and cell cycle genes including CDKN2B [93, 94]
Cyclin-dependent kinase 4 inhibitor B	*CDKN2B*	↓	Mediates cell cycle arrest at G1 [95]

**↑** = up-regulated, **↓** = down-regulated

## Discussion

Global expression studies have documented many differentially expressed genes in human prostate cancer [7, 9, 13–15, 96–102]. Lucas and Heath compiled a list of differentially expressed genes with reported prognostic significance in prostate cancer [30]. Of the 22 genes listed, 19 were differentially expressed in our TCGA dataset and there was agreement in expression pattern between 12 genes. *PTEN*, *TMPRSS2*, *MYC*, *SMAD4*, *EZH2*, *p53*, *BCL2*, *NPY*, *PLA2G7*, *Ki-67*, *p16*, and *BAX* expression in our findings matched what was presented in the literature. *PTEN*, a tumor suppressor, was down-regulated in malignant samples. The deletion of *PTEN* correlates with higher Gleason grade, risk of progression, and recurrence after therapy, and advanced localized or metastatic disease and death [103, 104]. *SMAD*4 was down-regulated in our TCGA prostate cancer data and has also been found to be down-regulated in prostate cancers, including advanced tumors [105, 106]. The deletion of this gene has led to invasive, metastatic, and lethal prostate cancers in a mouse model [39]. *TMPRSS2* was up-regulated and this is in agreement with reports of it being more highly expressed in prostate carcinoma compared to normal prostate epithelium [107, 108]. *TMPRSS2* contributes to the invasion and metastasis of prostate cancer [109]. Further, *TMPRSS2-ERG* gene fusion holds promise as a potential prostate cancer biomarker [110]. *MYC* was also up-regulated in this dataset and the overexpression (gene amplification, mRNA, and protein increase) of *MYC* in prostate cancer is well-documented [111–115]. *MYC* gene amplification was found more often in metastases [116, 117] and also correlated with poor prognostic factors like higher Gleason and histopathological scores [118], or greater chance of PSA recurrence [114]. *EZH2* up-regulation is reported here and in the literature where such overexpression led to increased proliferation in prostate cells and is associated with aggressive disease and increased risk of recurrence [119]. The expression of *p53* mRNA was increased in malignant samples in our TCGA data. In a study of prostate cancer patients, *p53* positive expression was seen in the majority (69.1%) of patients with the number of positive patients increasing as stage and Gleason score increased. P53 was also an independent predictor of recurrence [120]. *BCL2* mRNA expression was decreased in TCGA tumor samples. The absence of BCL-2 protein expression is reported in prostate cancer [120, 121]. Furthermore, BCL-2 expression is negative in androgen-dependent, but increased in hormone insensitive prostate cancers [122–124] and correlated with poor prognosis [125]. Pro-neuropeptide Y was up-regulated in this study and in the literature [126, 127]. Pro-neuropeptide Y up-regulation is associated with non-aggressive tumors [128] and regulates proliferation in prostate cancer cell lines [129]. *PLA2G7* was up-regulated in our data. It is reported to be more highly expressed in prostate cancer compared to benign samples [130, 131] and the TCGA samples studied here. Levels of *Ki-67* mRNA were increased in tumor versus non-malignant samples from our TCGA data and in the literature compared to normal tissue [132]. Furthermore Ki-67 protein is increased in prostate cancer [133–136], prostate cancer metastases [137, 138] and is a useful prognostic marker [139]. In our list of DEGs, *p16* was up-regulated. Recently, p16 expression was found in a large majority of prostate tissues [140]. *BAX* mRNA expression was increased in this TCGA dataset and BAX protein had increased expression in prostate cancer [141].

The remaining 7 DEGs in common with Lucas and Heath’s list displayed a discrepancy in expression pattern between our results and the literature. *TGF-β1* was not differentially expressed but *TGF-β2* was down-regulated. Expression of *TGF-β1* and *TGF-β2* was increased in prostate cancer compared to normal or non-malignant tissues [142–147]. However, *TGF-β3* was down-regulated in agreement with other reports of *TGF-β3* expression in prostate cancer [97, 148]. Both α and β isoforms of *IL-1* and *IL-6* were down-regulated in this TCGA dataset. *IL-1α* and *IL-6* were up-regulated in prostate cancer samples [149–153]. *IL-6* stimulated growth of LNCaP cells [154] and elevated *IL-6* was also associated with poor prognosis in prostate cancer [149, 155–162]. *IL-1β* has been reported both up- and down-regulated in the literature. Protein expression in patient samples was down-regulated [163] but elevated gene and protein expression in human cancer cells and tumors has also been reported [164]. In our list of DEGs, *p21* was down-regulated. Aaltomaa *et al*. reported p21 protein expression in the majority of prostate tumors but not in normal prostate epithelial cells [165] but other studies reported p21 immunostaining in only 20%-35% of cancer samples [166, 167]. Both *p21* and *p16* inhibited growth in prostate cancer cell lines [168]. Vascular endothelial growth factor A (*VEGF-A*) was down-regulated in our data. High expression of *VEGF* correlated with poor prognosis [169], but some studies reported that the higher expression of *VEGF-A* correlated with better clinical outcome [170]. *TRAIL/TNFSF10* was up-regulated in our TCGA data. While epithelial expression of TRAIL protein was stronger in tumors, stromal expression of TRAIL was decreased or absent in tumors [171, 172]. Only stromal TRAIL expression correlated with recurrence-free survival [171]. *NFKB1* was down-regulated in our data. However, *NFKB1* protein expression progressively increased in normal, benign prostatic hyperplasia and prostate cancer tissues [173]. The other DEGs with prognostic significance in prostate cancer that were not differentially expressed in our list of DEGs include *IL-7*, *CCL-2*, and *CDH1*.

Comparisons between the DEGs presented herein and DEGs listed in other studies highlight variance from experiment to experiment. Despite such variance a strong underlying correlation between datasets may still sometimes be seen [174]. These correlations would most likely be captured in a pathway approach. Thus our TCGA data was subject to pathway analysis. We found the “Ran regulation of mitotic spindle formation” pathway to be most significant in prostate cancer and the “TGF-β signaling” pathway to be activated in prostate cancer. Additionally, the following pathways were significant across both GSEA and SPIA methods and were associated with the non-malignant samples and were inhibited in the tumor samples: “proteoglycans in cancer”, “Hippo signaling pathway”, “cGMP-PKG signaling pathway”, “Ras signaling pathway”, “MAPK signaling pathway”, “Focal adhesion”, “Regulation of actin cytoskeleton”, “Oxytocin signaling pathway”, and “Pathways in cancer”.

### Ran regulation of mitotic spindle formation pathway

Ran is a small GTPase of the Ras family known to function in directing nucleocytoplasmic transport, in cell cycle control through regulation of transition to S-phase and mitosis, and in regulating the mitotic spindle during mitosis and the reassembly of the nuclear envelope after mitosis [175]. Ran’s control over the mitotic spindle is the pathway that was shown to be significant in prostate cancer in our data. Proper functioning of this pathway assembles spindle microtubules for chromosome alignment and segregation in a way that ensures a single copy of each chromosome is distributed to the daughter cells, thus avoiding aneuploidy [74, 75, 176]. Each of the genes in this pathway, which include Ran, its regulators, accessory proteins, spindle assembly factors, and import/export factors, was up-regulated (Table 6). Ran’s function in mitotic spindle assembly is reviewed by Clarke and Zhang [176]. Ran-GDP is converted to Ran-GTP by the guanine nucleotide-exchange factor RCC1 and is hydrolyzed back to Ran-GDP with the aid of the GTPase activating protein RanGAP1 and RanBP1/2. The specific localization of RCC1 and RanGAP1/RanBP2 results in the formation of Ran-GTP at precise points along spindle assembly. Importin-α/importin-β complexes carry spindle assembly factors such as TPX2 and NuMA into the nucleus where they are released at chromosomes after interaction with Ran-GTP. Spindle assembly factors then interact with other molecules such as Aurora kinase A to form the spindle.

Ran-GTP overexpression was reported in various human cancers [177–181] and multiple cancer cell lines [181, 182]. Ran proved critical for epithelial ovarian cancer cell survival [183] and its overexpression caused malignant transformation in rat mammary cells [184]. Silencing of Ran in tumor cell lines, but not normal cells, led to cell death after aberrations in mitotic spindle assembly and mitochondrial function [181]. In agreement with our data, other pathway components and Ran-associated genes are also overexpressed in cancer: Aurora kinase A [185], TPX2 [186–188], and HSET [189]. Ran has not been extensively studied in prostate cancer. There are reports of increased Ran expression in prostate tumor tissues [190] and Ran functions as an androgen receptor coactivator [191, 192].

### TGF-β Signaling

The TGF-β signaling pathway was activated in the malignant condition in this TCGA prostate cancer dataset. In general, TGF-β signaling regulates cell proliferation, migration, differentiation, epithelial-mesenchymal transition, immune-suppression, and apoptosis [85, 193]. Several components of the TGF-β signaling pathway were differentially expressed (Table 8). The binding of active TGF-β to its receptors begins a phosphorylation cascade that activates Smad transcription factors which translocate to the nucleus. In the nucleus, the Smad complex binds various transcription factors, coactivators, co-repressors, and chromatin remodeling factors to regulate gene expression [194, 195]. Ultimately, TGF-β signaling promotes expression of inhibitors of cell cycle progression and suppresses proliferative genes [195, 196]. Tumor cells can subvert the suppressive effect of TGF-β signaling seen in normal cells to promote tumorigenesis [194, 195].

Several studies have reported the increase of TGF-β isoforms in prostate cancer [142, 145–147, 197, 198], however our study shows a significant decrease in *TGF-β2* and *TGF-β3* gene expression and no differential expression of *TGF-β1*. Our results are, however, corroborated by the work of Dallas *et al*. which showed both latent and active forms of TGF-β2 were decreased in malignant prostate cells compared to normal prostate epithelial cells cultured from the same patient [199]. Our results are also corroborated by studies showing lost or decreased expression of TGF-β3 [143, 148]. In our TCGA dataset, all three TGF-β receptors were down-regulated. Loss of TGF-β receptors is consistent with literature [146, 200–204] and represents a mechanism through which tumors avoid growth suppression by TGF-β, thus facilitating the development of cancer after oncogenic triggers [195]. Additionally, down-regulation of TGF-β1, β2, and β3 is associated with androgen-stimulated growth of prostate cancer cells [205].

Although TGF-β signaling typically operates through Smad proteins, the pathway signal may also be diverted through other Smad-independent pathways like PI3K/AKT, ERK/MAPK, JNK/p38 MAPK and Rho-like GTPase signaling pathways [151, 206]. Since Smad genes were down-regulated, we looked at other effectors and found serine/threonine-protein phosphatase 2A 65 kDa regulatory subunit A alpha and beta isoforms (*PPP2R1A*, *PPP2R1B*) to be up-regulated along with the targets ribosomal protein S6 kinase β-1 and β-2 (*RPS6KB1*, *RPS6KB2*), the serine/threonine-protein phosphatase 2A catalytic subunit β isoform (*PPP2CB*) was down-regulated, both *RhoA* and *ROCK1* were down-regulated and *MAPK1* and *MAPK3* were also down-regulated. In our TCGA data, MAPK signaling pathway was significantly different between tumor and non-malignant samples, however it was more associated with non-malignant samples whereas TGF-β was more associated with tumor samples. Erk signaling alters the expression of genes controlling cell motility, and cell-matrix adhesion and interactions [207]. Cell motility and cell-matrix adhesion-related gene sets were also significantly enriched in the non-malignant samples of our TCGA prostate cancer data (Table 5).

### Pathway Comparison

There were a few surprising results from GSEA analysis—namely, the significance of prolactin and oxytocin signaling pathways and thyroid hormone synthesis pathway. The genes contributing to the enrichment of these pathways in non-malignant samples were not the namesake hormones themselves, but the multiple kinases, phosphatases, and calcium or potassium channel proteins that participate in hormone signaling (S10–S12 Tables). In the case of oxytocin signaling, the pathway operates through both calcium signaling and MAPK signaling (S1 Fig), which were also found to be significant. For the prolactin pathway (S2 Fig), the enrichment of MAPK kinases and PI3K kinases is abundant however prolactin itself is not enriched (S11 Table). Finally, for thyroid synthesis pathway (S3 Fig), none of the hormones or receptors are present but other components through which they operate are (S12 Table). Thus it appears these pathways could have been flagged due to substantial overlap with the signaling of other pathways since neither oxytocin, prolactin, or thyroid stimulating hormone nor their receptors were differentially expressed. These results demonstrate the limitation of GSEA discussed previously, the topology of genes in the pathways is unaccounted for. SPIA is a complementary pathway method that does consider the position of genes in the pathway. It is noteworthy that SPIA analysis was able to filter prolactin and thyroid hormone synthesis pathways from significant results.

Comparison to previous pathway studies which used microarray data or single nucleotide polymorphisms from genome-wide association studies showed that several pathways were identified across experimental platforms. Savli *et al*. looked at gene networks and pathway analysis in prostate cancer [208]. However, that study used microarray to measure gene expression and found 738 up-regulated genes and 515 down-regulated genes. This study used RNA sequencing data and found 5,736 up-regulated genes and 5,379 down-regulated genes. Some advantages of a sequencing method over microarray approach include more extensive transcript identification beyond the coverage of sequence libraries although correlation between some sequencing approaches and microarray platforms has been demonstrated [34]. Additionally, our patient pool was much larger (173 versus 21 tumor and 52 versus 10 non-malignant). The methods for identifying pathways was also different. Savli *et al*. used Ingenuity Pathway Analysis to identify pathways and construct gene networks. “Axonal guidance signaling” (down-regulated) and “acute phase response” (up-regulated) were the most significant pathways among the up- and down-regulated canonical pathways reported by Savli *et al*. but were not found in this study’s results. However other important pathways in prostate cancer were found in both studies including “actin cytoskeleton” (down-regulated in both), “calcium signaling” (up-regulated in Savli *et al*., down-regulated in ours), and “MAPK signaling” (down-regulated in both). Jia *et al*. used a combination of GSEA and Plink set-based tests on microarray data and genome-wide association studies to identify thirteen KEGG pathways involved in prostate cancer [209]. In this study, we found five of these KEGG pathways to be important in prostate cancer: regulation of actin cytoskeleton, small cell lung cancer, cell cycle, chronic myeloid leukemia, and TGF-β signaling pathway.

## Conclusion

This work presents a comprehensive gene expression profile of human prostate cancer. Differential gene expression was analyzed in the context of gene sets and pathways to identify signature pathways associated with prostate cancer. “TGF-β signaling” and “Ran regulation of mitotic spindle formation” pathways were strongly associated with prostate cancer. Since it is an underexplored area in prostate cancer, we suggest Ran pathway components for further investigation in prostate cancer pathogenesis. Several other significant pathways confirm reported findings from microarray data that suggest actin cytoskeleton regulation, cell cycle, MAPK signaling, and calcium signaling are also altered in prostate cancer. We further observed that none of the most highly altered genes with the largest increases or decreases in expression appeared in the significant pathways. Thus we have demonstrated that both differential expression and pathway analysis are important in extracting meaningful information.

## Supporting Information

S1 Fig KEGG Oxytocin Signaling Pathway. Differentially expressed genes are highlighted in red.
(PNG)

S2 Fig KEGG Prolactin Signaling Pathway. Differentially expressed genes are highlighted in red.
(PNG)

S3 Fig KEGG Thyroid Hormone Synthesis Pathway. Differentially expressed genes are highlighted in red.
(PNG)

S1 Table Age- and stage matched human prostate cancer mRNA expression dataset. This file includes the RNAseq expression data for the 225 age- and stage-matched prostate cancer non-malignant and tumor samples downloaded from The Cancer Genome Atlas and used for the analyses presented in this work.
(XLSX)

S2 Table Selected KEGG pathways used for all pathway analyses. This is the set of KEGG pathways used in Gene Set Enrichment Analysis and Signaling Pathway Impact Analysis. Pathways likely to have little relevance to prostate cancer (*e*.*g*. parasitic, bacterial, and viral infectious diseases, substance dependencies, and specific immune, neurodegenerative, and cardiovascular diseases) have been excluded.
(XLSX)

S3 Table Differentially expressed genes in human prostate cancer. A total of 11,115 genes and transcripts were differentially expressed according to DESeq2 analysis using Wald statistics. All statistical parameters plus the calculated log_2_ fold change are presented.
(XLSX)

S4 Table Complete DESeq2 analysis results including all genes and transcripts evaluated. Complete results of DESeq2 analysis with statistical parameters and calculated log_2_ fold change.
(XLSX)

S5 Table Classification of differentially expressed genes by protein class and gene ontology. Complete classification based on PANTHER protein class, GO Molecular Function and GO Biological Process terms.
(XLSX)

S6 Table PANTHER overrepresentation results for protein class and gene ontology. This is the full pathway overrepresentation analysis of protein class and GO Biological Process categories among DEGs from the dataset.
(XLSX)

S7 Table Complete GSEA results for BioCarta, Reactome and KEGG gene sets. (XLSX)

S8 Table Genes and Transcripts contributing to KEGG Calcium Signaling Pathway enrichment in GSEA. These genes and transcripts from the evaluated TCGA dataset contribute to the enrichment of the KEGG Calcium Signaling Pathway in non-malignant samples.
(XLSX)

S9 Table Complete SPIA results for KEGG pathways. (XLSX)

S10 Table Genes contributing to KEGG Oxytocin Signaling Pathway enrichment. These genes and transcripts from the evaluated TCGA dataset contribute to the enrichment of the KEGG Calcium Signaling Pathway in non-malignant samples.
(XLSX)

S11 Table Genes contributing to KEGG Prolactin Signaling Pathway enrichment. These genes and transcripts from the evaluated TCGA dataset contribute to the enrichment of the KEGG Calcium Signaling Pathway in non-malignant samples.
(XLSX)

S12 Table Genes contributing to KEGG Thyroid Hormone Synthesis Pathway enrichment. These genes and transcripts from the evaluated TCGA dataset contribute to the enrichment of the KEGG Calcium Signaling Pathway in non-malignant samples.
(XLSX)

## Abbreviations

PSA: prostate-specific antigen
DEGs: differentially expressed genes
MAPK: mitogen activated protein kinase
TGF-β: transforming growth factor-beta
TCGA: The Cancer Genome Atlas
FDR: false discovery rate
PANTHER: Protein ANalysis THrough Evolutionary Relationships
GO: Gene Ontology
GSEA: Gene Set Enrichment Analysis
KEGG: Kyoto Encyclopedia of Genes and Genomes
SPIA: Signaling Pathway Impact Analysis
ES: enrichment score
NES: normalized enrichment score
pNDE: probability of overrepresentation
pPERT: probability of perturbation
