# Supplementary figures and images for: Differentially Expressed Genes and Signature Pathways of Human Prostate Cancer
Source: PLoS One. 2015 Dec 18;10(12):e0145322. doi: 10.1371/journal.pone.0145322 (PMC4687717; doi:10.1371/journal.pone.0145322)

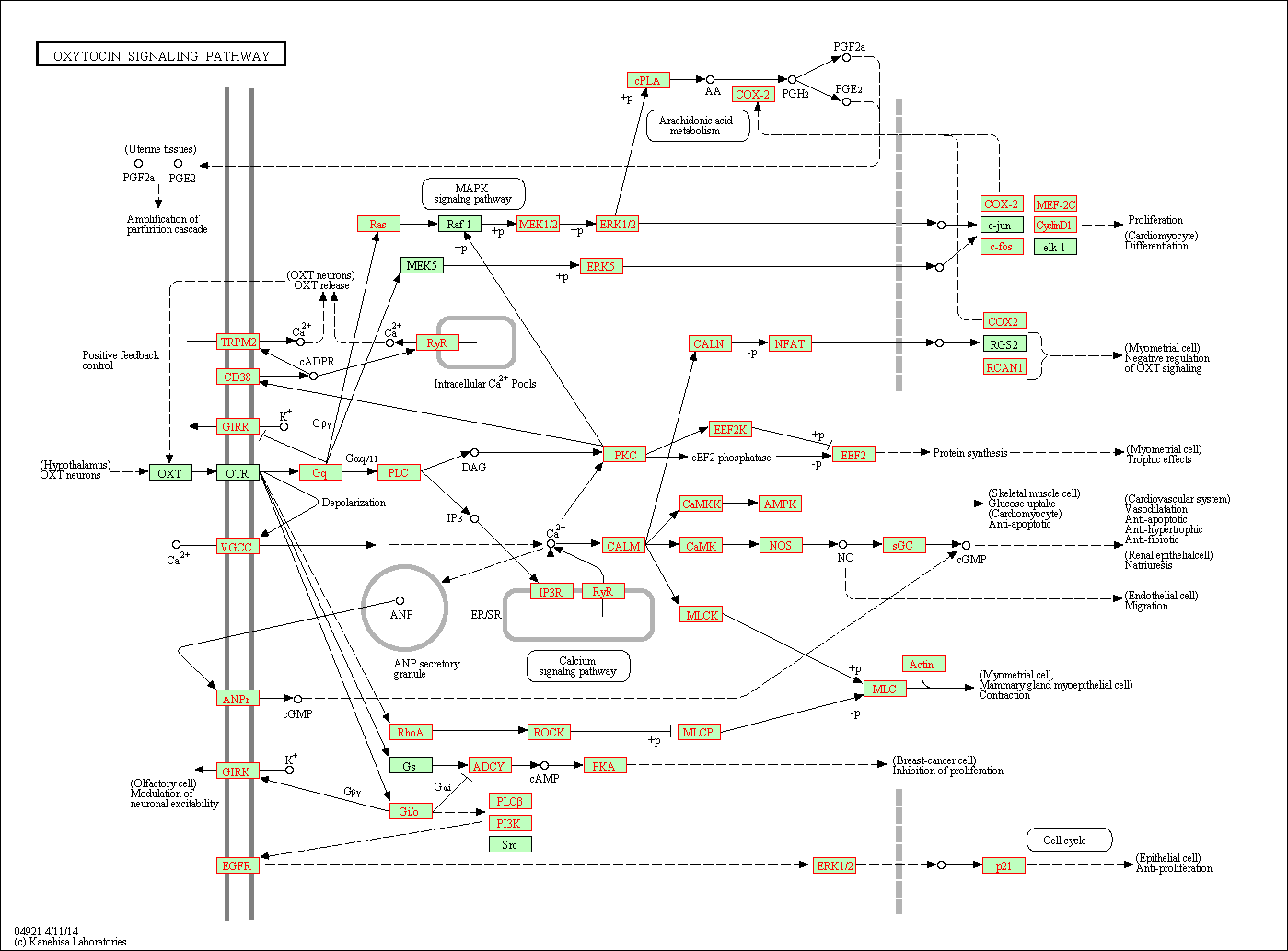

Supplement: S1 Fig — Differentially expressed genes are highlighted in red. (PNG) [file pone.0145322.s001.png]

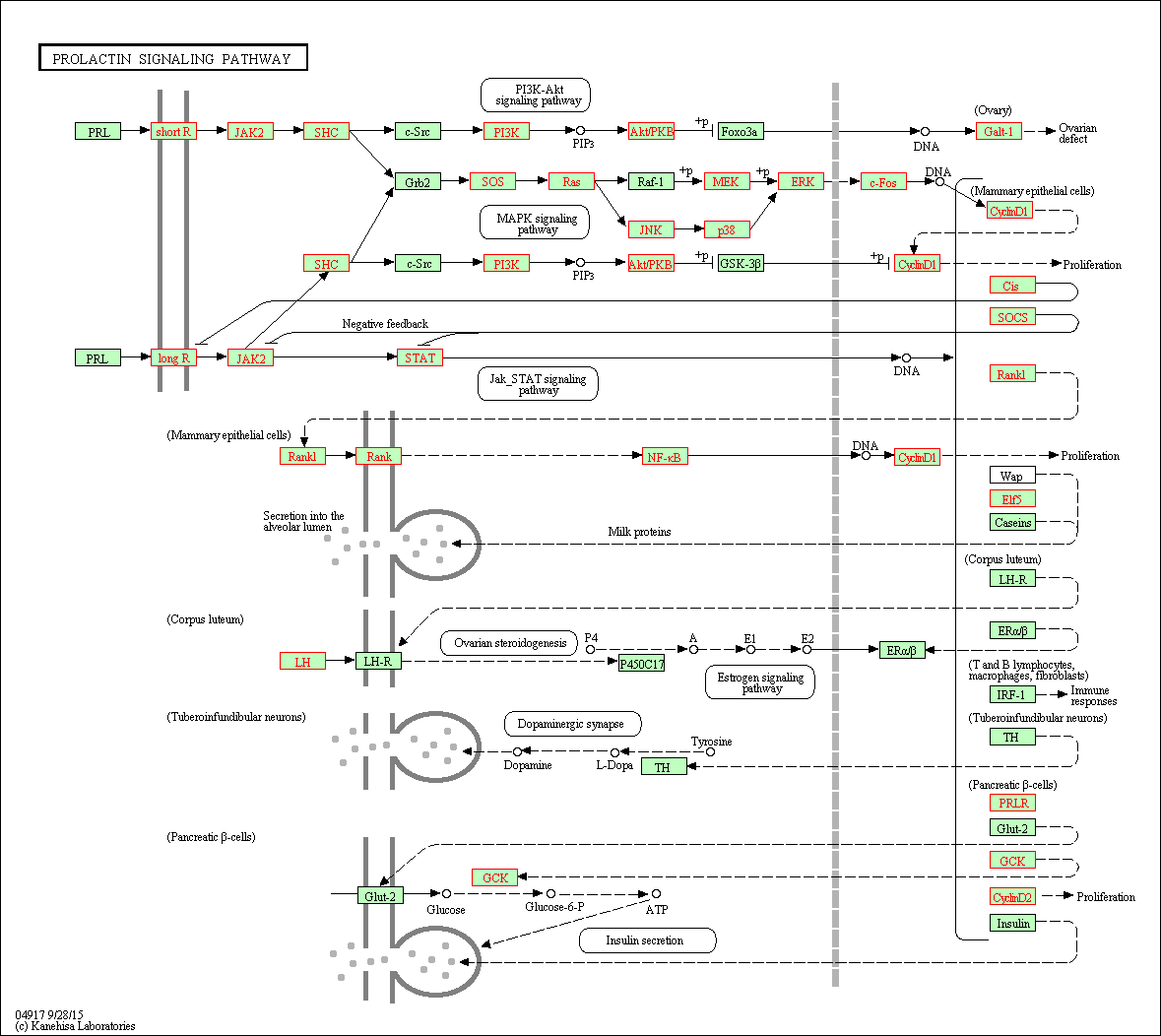

Supplement: S2 Fig — Differentially expressed genes are highlighted in red. (PNG) [file pone.0145322.s002.png]

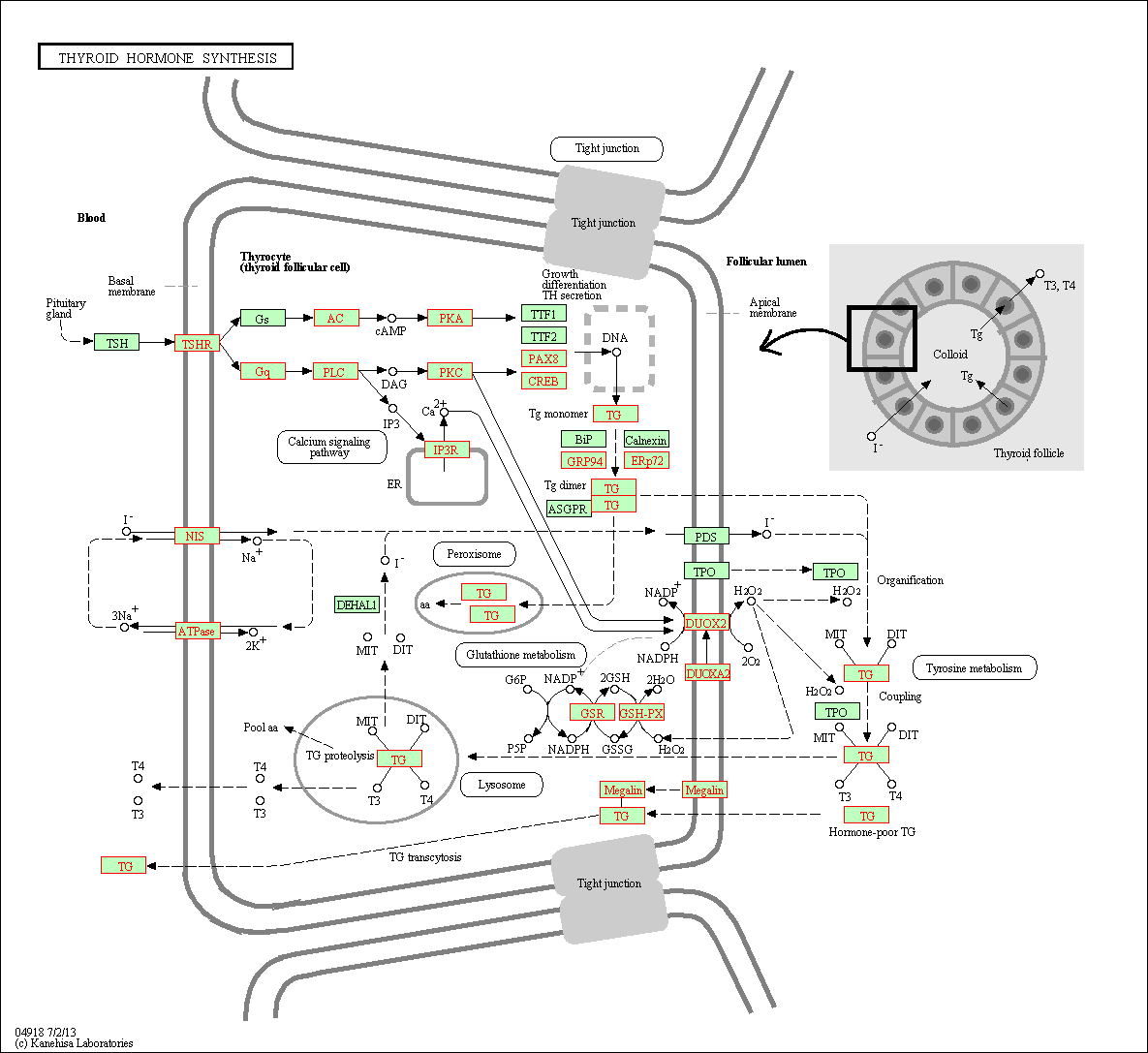

Supplement: S3 Fig — Differentially expressed genes are highlighted in red. (PNG) [file pone.0145322.s003.png]
